# Supplementary material for: Sorting at embryonic boundaries requires high heterotypic interfacial tension
Source: Nat Commun. 2017 Jul 31;8:157. doi: 10.1038/s41467-017-00146-x (PMC5537356; doi:10.1038/s41467-017-00146-x)
Supplement: Supplementary file 2 — Supplementary Software 1 [file 41467_2017_146_MOESM2_ESM.zip › PottsModel/SrcPottsModel/doc/model/Cell.html]

Cell


JavaScript is disabled on your browser.


Skip navigation links


- Overview
- Package
- Class
- Use
- Tree
- Deprecated
- Index
- Help

- Prev Class
- Next Class

- Frames
- No Frames

- All Classes

- Summary:
- Nested |
- Field |
- Constr |
- Method

- Detail:
- Field |
- Constr |
- Method


model

## Class Cell

- java.lang.Object
- - mvc.AObservable
  - - model.Cell

- All Implemented Interfaces:
  :   KnowsConstants, IObservable

  Direct Known Subclasses:
  :   SquareCell

  ---

    

  ```
  public class Cell
  extends AObservable
  implements KnowsConstants
  ```

- - ### Nested Class Summary

    Nested Classes

    | Modifier and Type | Class and Description |
    | --- | --- |
    | `static class` | `Cell.CellType` |
    | `static class` | `Cell.Energy` |
    | `static class` | `Cell.State` Enum class representing the different states of the cell that can be passed as a notification message to the different class observers. |
  - ### Field Summary

    Fields

    | Modifier and Type | Field and Description |
    | --- | --- |
    | `Constants` | `aConstants` |
    | `double` | `cAreaUnit` |
    | `double` | `cEdgeUnit` |
    | `static java.lang.String` | `cNotificationSource` |
  - ### Constructor Summary

    Constructors

    Constructor and Description

    `Cell(Cell.CellType pType,
    int pID,
    Lattice pLattice)`

    `Cell(Cell pCell)`

    Copy constructor returning a copy of the passed BaseCell with identical
    member variables including a shallow copy of the pixelList.

    `Cell(int pID,
    Lattice pLattice)`
  - ### Method Summary

    All Methods Static Methods Instance Methods Concrete Methods

    | Modifier and Type | Method and Description |
    | --- | --- |
    | `void` | `addPixel(Pixel pPixel)` Add Pixel to Cell. |
    | `boolean` | `equals(Cell pOther)` |
    | `double` | `getArea()` |
    | `double` | `getAreaEnergy()` Returns area energy such that: energy = areaElasticity \* (area - targetArea)^2 If any of targetArea or area are negative, then returns 0. |
    | `java.awt.Color` | `getCellTypeColor()` |
    | `static java.awt.Color` | `getCellTypeColor(Cell.CellType pType)` |
    | `static java.lang.String` | `getCellTypeColorName(Cell.CellType pType)` |
    | `static Cell.CellType` | `getCellTypeFromID(int pID)` |
    | `Coordinates` | `getCenterOfMass()` |
    | `double` | `getComX()` |
    | `double` | `getComY()` |
    | `Constants` | `getConstants()` |
    | `CellDisplay` | `getDisplay()` |
    | `double` | `getDistanceToCell(Cell pCell)` |
    | `int` | `getID()` |
    | `java.lang.String` | `getInformation()` |
    | `double` | `getInteractionEnergy()` |
    | `double` | `getIsoperimetricQuotient()` |
    | `static double` | `getLocalAreaEnergy(Cell pCell1, Cell pCell2)` The sum of the area-dependent energies. |
    | `static double` | `getLocalPerimeterEnergy(Cell pCell1, Cell pCell2)` The sum of the perimeter-dependent energies. |
    | `CellSet` | `getNeighborCells()` |
    | `java.lang.String` | `getNotificationSource()` |
    | `double` | `getPerimeter()` |
    | `double` | `getPerimeterEnergy()` Returns perimeter energy such that: energy = perimeterElasticity \* (area - targetPerimeter)^2 If any of targetPerimeter or perimeter are negative, then returns 0. |
    | `Pixel` | `getPixel(int x, int y)` |
    | `double` | `getShapeMetric()` |
    | `Cell.CellType` | `getType()` |
    | `int` | `hashCode()` |
    | `void` | `initalize()` |
    | `boolean` | `isIsolated()` |
    | `boolean` | `isIsolated(CellSet pNeighborCells)` |
    | `boolean` | `isMedium()` |
    | `void` | `removePixel(Pixel pPixel)` Removes pixel from cell and readjusts center of mass, area and perimeter if it's not medium. |
    | `java.lang.String` | `toString()` |
    | `void` | `updateParameters(Pixel pPixel, boolean pIsAdded)` Call after a pixel has been added or removed from a cell |

    - ### Methods inherited from class mvc.AObservable

      `addObserver, getNotificationSequenceNumber, notifyObservers, removeAllObservers, removeObserver`
    - ### Methods inherited from class java.lang.Object

      `equals, getClass, notify, notifyAll, wait, wait, wait`

- - ### Field Detail


    - #### cNotificationSource

      ```
      public static final java.lang.String cNotificationSource
      ```

      See Also:
      :   Constant Field Values


    - #### aConstants

      ```
      public final Constants aConstants
      ```


    - #### cAreaUnit

      ```
      public final double cAreaUnit
      ```

      See Also:
      :   Constant Field Values


    - #### cEdgeUnit

      ```
      public final double cEdgeUnit
      ```
  - ### Constructor Detail


    - #### Cell

      ```
      public Cell(Cell.CellType pType,
                  int pID,
                  Lattice pLattice)
      ```


    - #### Cell

      ```
      public Cell(int pID,
                  Lattice pLattice)
      ```


    - #### Cell

      ```
      public Cell(Cell pCell)
      ```

      Copy constructor returning a copy of the passed BaseCell with identical
      member variables including a shallow copy of the pixelList.

      Parameters:
      :   `c` - the ACell to copy
  - ### Method Detail


    - #### initalize

      ```
      public void initalize()
      ```


    - #### getCellTypeColor

      ```
      public static java.awt.Color getCellTypeColor(Cell.CellType pType)
      ```


    - #### getCellTypeColorName

      ```
      public static java.lang.String getCellTypeColorName(Cell.CellType pType)
      ```


    - #### getCellTypeColor

      ```
      public java.awt.Color getCellTypeColor()
      ```


    - #### getDisplay

      ```
      public CellDisplay getDisplay()
      ```


    - #### getPixel

      ```
      public Pixel getPixel(int x,
                            int y)
      ```


    - #### addPixel

      ```
      public void addPixel(Pixel pPixel)
      ```

      Add Pixel to Cell. Center of mass, area and perimeter are not adjusted if
      it's medium.

      Parameters:
      :   `pPixel` - the APixel to add to this Cell


    - #### updateParameters

      ```
      public void updateParameters(Pixel pPixel,
                                   boolean pIsAdded)
      ```

      Call after a pixel has been added or removed from a cell

      Parameters:
      :   `pIsAdded` -


    - #### getNeighborCells

      ```
      public CellSet getNeighborCells()
      ```


    - #### isIsolated

      ```
      public boolean isIsolated()
      ```


    - #### isIsolated

      ```
      public boolean isIsolated(CellSet pNeighborCells)
      ```


    - #### removePixel

      ```
      public void removePixel(Pixel pPixel)
      ```

      Removes pixel from cell and readjusts center of mass, area and perimeter
      if it's not medium.

      Parameters:
      :   `pPixel` - the Pixel to remove from this Cell


    - #### getAreaEnergy

      ```
      public double getAreaEnergy()
      ```

      Returns area energy such that: energy = areaElasticity \* (area -
      targetArea)^2 If any of targetArea or area are negative, then returns 0.

      Returns:
      :   the area energy of the cell


    - #### getPerimeterEnergy

      ```
      public double getPerimeterEnergy()
      ```

      Returns perimeter energy such that: energy = perimeterElasticity \* (area
      - targetPerimeter)^2 If any of targetPerimeter or perimeter are negative,
      then returns 0.

      Returns:
      :   the perimeter energy of the cell


    - #### getInteractionEnergy

      ```
      public double getInteractionEnergy()
      ```


    - #### getCellTypeFromID

      ```
      public static Cell.CellType getCellTypeFromID(int pID)
      ```


    - #### equals

      ```
      public boolean equals(Cell pOther)
      ```


    - #### hashCode

      ```
      public int hashCode()
      ```

      Overrides:
      :   `hashCode` in class `java.lang.Object`


    - #### toString

      ```
      public java.lang.String toString()
      ```

      Overrides:
      :   `toString` in class `java.lang.Object`


    - #### getInformation

      ```
      public java.lang.String getInformation()
      ```


    - #### getDistanceToCell

      ```
      public double getDistanceToCell(Cell pCell)
      ```


    - #### getCenterOfMass

      ```
      public Coordinates getCenterOfMass()
      ```


    - #### isMedium

      ```
      public boolean isMedium()
      ```


    - #### getID

      ```
      public int getID()
      ```


    - #### getType

      ```
      public Cell.CellType getType()
      ```


    - #### getPerimeter

      ```
      public double getPerimeter()
      ```


    - #### getArea

      ```
      public double getArea()
      ```


    - #### getComX

      ```
      public double getComX()
      ```


    - #### getComY

      ```
      public double getComY()
      ```


    - #### getLocalAreaEnergy

      ```
      public static double getLocalAreaEnergy(Cell pCell1,
                                              Cell pCell2)
      ```

      The sum of the area-dependent energies.

      Parameters:
      :   `pCell1` - the first cell
      :   `pCell2` - the second cell

      Returns:


    - #### getLocalPerimeterEnergy

      ```
      public static double getLocalPerimeterEnergy(Cell pCell1,
                                                   Cell pCell2)
      ```

      The sum of the perimeter-dependent energies.

      Parameters:
      :   `pCell1` - the first cell
      :   `pCell2` - the second cell

      Returns:


    - #### getNotificationSource

      ```
      public java.lang.String getNotificationSource()
      ```

      Specified by:
      :   `getNotificationSource` in interface `IObservable`

      Overrides:
      :   `getNotificationSource` in class `AObservable`


    - #### getShapeMetric

      ```
      public double getShapeMetric()
      ```


    - #### getConstants

      ```
      public Constants getConstants()
      ```

      Specified by:
      :   `getConstants` in interface `KnowsConstants`


    - #### getIsoperimetricQuotient

      ```
      public double getIsoperimetricQuotient()
      ```


Skip navigation links


- Overview
- Package
- Class
- Use
- Tree
- Deprecated
- Index
- Help

- Prev Class
- Next Class

- Frames
- No Frames

- All Classes

- Summary:
- Nested |
- Field |
- Constr |
- Method

- Detail:
- Field |
- Constr |
- Method
